# Management of Postoperative Seroma: Recommendations Based on a 12-Year Retrospective Study

**DOI:** 10.3390/jcm11175062

**Published:** 2022-08-28

**Authors:** Athanasios Papanikolaou, Eliane Minger, Michael-Alexander Pais, Mihai Constantinescu, Radu Olariu, Adriaan Grobbelaar, Ioana Lese

**Affiliations:** 1 Department of Plastic and Hand Surgery, Inselspital, Bern University Hospital, 3008 Bern, Switzerland; 2 Department for BioMedical Research, University of Bern, 3008 Bern, Switzerland

**Keywords:** seroma, management, indicators for revisional surgery

## Abstract

Introduction: Seroma formation is a serious postoperative complication. Since the management algorithms available in the literature are scarce, we aimed to analyze our experience with postoperative seroma in order to identify indicators for revisional surgery and propose recommendations for management. Methods: This retrospective study included all patients with postoperative seroma treated in a tertiary university hospital from 2008 to 2020. Patients’ demographics, medical history, and seroma treatment details were recorded and analyzed. Results: Overall, 156 patients were included: 41% were initially treated through needle aspiration, with 61% eventually undergoing surgical treatment for postoperative seroma. Comorbidities, such as heart failure and coronary heart disease, were significantly associated with an increased need for revisional surgery (*p* < 0.05). Both a duration of >40 days of repeated needle aspirations and drain re-insertions were significantly correlated with an increased risk for revisional surgery (*p* < 0.05). Conclusion: Patients requiring seroma aspiration should be counseled on surgical treatment sooner rather than later, as prolonged aspiration time (over 40 days) greatly increases the risk of surgical revision. Moreover, the reinsertion of a drain should only be used as a temporizing measure, at most, and patients requiring a drain to control the size of the seroma should promptly be scheduled for a surgical revision.

## 1. Introduction

Seroma formation is a commonly occurring complication following a great variety of surgical procedures. The spectrum ranges from self-limiting conditions to repetitive, therapy-resistant fluid collections. If untreated, functional impairment, oncologic treatment delays, and wound infections may cause added morbidity. In addition, the financial consequences of repetitive treatments and interventions are an added burden on the medical system.

Many clinical studies have tried to identify possible risk factors contributing to seroma formation, as well as strategies to minimize these risks. Preventive measures and surgical strategies have been proposed to reduce the incidence of seromas—especially after breast surgery, flap harvesting, abdominoplasty, and lymph node dissections—and include quilting and progressive tension sutures [1], sclerosing or adhesive substances, the application of triamcinolone or talc [2], as well as compression and immobilization [3,4,5,6,7]. Other studies have proposed comorbidities, such as increased BMI and hypertension, as possible risk factors in the development of seromas [8,9].

Treatment options in seroma management include serial aspirations; the application of sclerosing agents in the seroma cavity, such as doxycycline, bleomycin, or nanoparticles; surgical marsupialization or excision/debridement of the seroma capsule; and drainage. However, no single ideal treatment option has been proven to guarantee the therapeutic outcome, and most patients require frequent percutaneous aspirations in an outpatient setting until no further recurrence of seroma formation is clinically present [10,11]. Certainly, the risk of infection with every attempt at seroma aspiration must be considered and communicated to the patients accordingly. If an infection of the residual seroma occurs, an abscess formation will make surgical revision for evacuation unavoidable.

The aims of our study were to analyze the need and type of therapeutic interventions for postoperative seromas in our unit over the last 12 years and to identify indicators for revision surgery during the course of seromas in order to provide better management recommendations for the future.

## 2. Materials and Methods

This retrospective study included all patients treated in our unit from 2008 to 2020 that were diagnosed with postoperative seroma at the surgical site, regardless of whether the initial surgery that led to seroma was performed in our center or the patients were referred to us for further treatment after being operated elsewhere. Procedures that led to seroma formation at the surgical site included body-contouring procedures (bodylifts, abdominoplasties, and thigh lifts), breast surgery (augmentation, reconstruction, and reduction), soft tissue infections, lymph node biopsies and dissections, donor sites of flaps harvested for reconstructive surgery, and others. Patients with lymphatic vessel involvement diagnosed by means of lymphoscintigraphy were excluded from this study, as the reason for developing a fluid collection at the surgical site in such cases was mostly due to lymphatic fistula and not true postoperative seroma formation. The following patient-related data were collected: age, gender, body mass index (BMI), regular consumption of alcohol or nicotine, American Society of Anesthesiologists (ASA) score, diabetes, high blood pressure, heart failure/coronary disease, cerebrovascular disease, peripheral arterial occlusive disease, chronic venous insufficiency, chemotherapy, and radiotherapy. Furthermore, we assessed treatment-specific variables, including the application of seroma preventive measures, the duration of the seroma, the anatomic location of the seroma, the number of fluid aspirations performed, the need for drain re-insertion, the time period for leaving the drains in place at the time of the original surgery, wound-related readmissions, the duration of the multiple aspirations, the need for surgical revision of the seroma, and seroma recurrence. The minimum follow-up period was at least 1 year.

The indication for surgical revision was specified in cases where the seromas presented signs of infection or skin necrosis. Moreover, in patients undergoing aspirations, when the aspirated volume did not seem to decrease after 5–6 aspirations, patients were given the choice of revisional surgery.

Statistical analyses were performed using Prism 8.0 (GraphPad Software, San Diego, CA, USA). Student’s t-test or the Mann–Whitney test, depending on the distribution of the data, was used for continuous variables (age, BMI, volumes, and variables expressed in days), while the Pearson’s X^2^ and Fischer’s test were employed for the categorical variables in order to conduct comparisons between patients with and without aspirations, as well as between patients with and without revisional surgery.

## 3. Results

**Figure 1:**
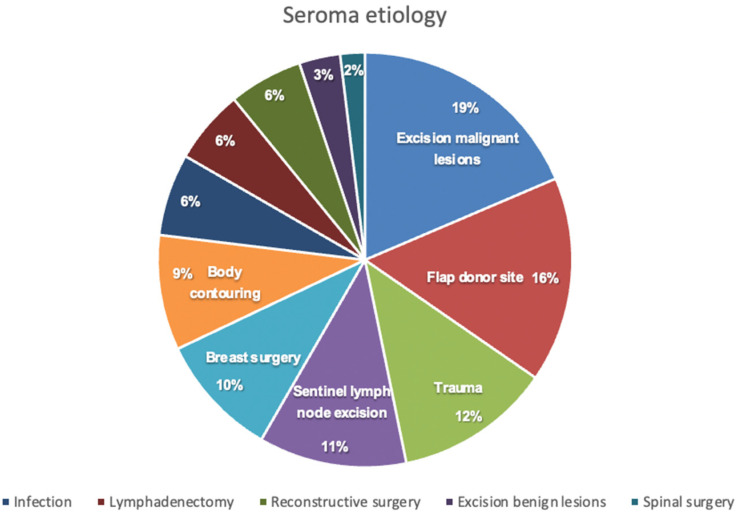
Seroma etiology.

We assessed a total of 156 patients that underwent treatment for postoperative seroma in our clinic. The etiology of seroma formation consisted of a wide spectrum of surgical procedures, as depicted in Figure 1, with the excision of malignant lesions and the donor site of harvested flaps for reconstructive surgery being the most common. Patients` characteristics are outlined in Table 1 and Table 2.

**Figure 2:**
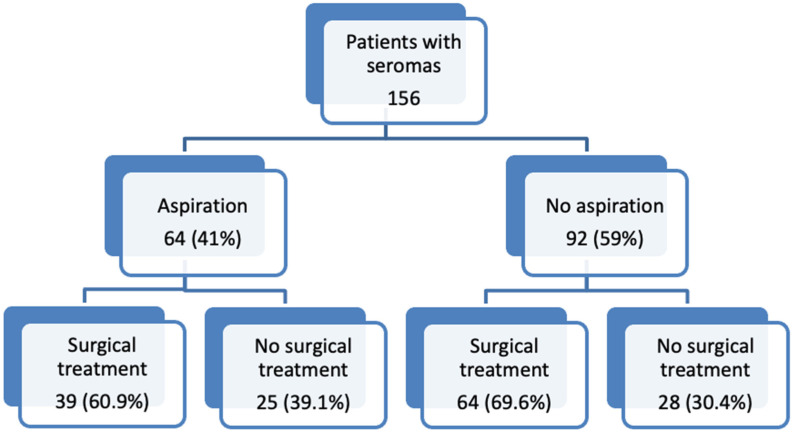
Seroma treatment.

Of all the treated patients, 103 patients (66%) had to undergo surgery for definitive therapy of seroma and 53 (34%) were treated non-surgically, as outlined in Figure 2. From the group of patients that underwent seroma aspirations, 39 patients (60.9%) needed surgical treatment and 25 (39.1%) were managed without surgical intervention. Out of the 92 patients who did not undergo aspiration, 64 (69.6%) required surgery: Ten patients underwent reinsertion of the drain since the volume of seroma detected through imaging was approximately over 300 mL; however, the reinsertion of the drain could not treat the seroma and the patients needed surgical revision in the end. A total of 25 patients presented with infection, and 17 patients already showed signs of skin necrosis at presentation; therefore, the indication for surgery could not be avoided anymore. The remaining 10 patients refused conservative therapy from the beginning.

The median number of surgeries per operated patient was two, ranging from one to eight. Infection was the reason for revision surgery among 14 patients.

The surgical procedures were divided into primary definitive treatment, when the wound was closed during the same operation as the seroma excision and debridement, and delayed closures, as outlined in Table 3. Additionally, when more than one surgery was needed, the wound was, in the end, closed secondarily over the drains in 39 patients. Skin grafts were used in six patients, while a flap was necessary to close the defect in another six patients. Negative pressure wound therapy (NPWT) was applied in 51 patients, with the median duration of NPWT being 10 days and ranging from 3 to 123 days.

Upon examining the cohort of patients that underwent revisional surgery for seromas, we found no differences between the patients that underwent surgery for seroma treatment and the ones who did not in regards to BMI, age, the total output of the drains, the number of aspirations, or the total volume of aspirations. Among all the comorbidities, only heart failure and the presence of coronary heart disease showed an association with the need for revisional surgery (*p* < 0.05). Moreover, there was a statistically significant difference in seroma duration between these groups: patients that did not undergo surgery had a median duration of seroma of 28 days (range 7–305 days), while the patients that underwent surgical treatment had a median duration of seroma of 58 days (range 3–1218 days) (*p* = 0.0124).

When examining the cohort of patients that underwent aspirations in regards to the timepoint of qualifying for surgical revision, we found that surgery ensued in only 31.8% of the patients that underwent aspirations for up to 40 days, while 81.5% of the patients requiring seroma aspiration for over 40 days eventually needed revision surgery (*p* = 0.001). Specifically, within the group of patients that needed aspirations for >40 days, these patients had a 9.43 times higher risk of requiring an operation as a definitive treatment of refractory seroma compared with the patients that underwent seroma aspirations for less than 40 days. Moreover, when looking at the patients requiring seroma aspiration, surgery was eventually necessary for all patients where the reinsertion of a drain was indicated (*p* = 0.018).

However, of all 156 patients treated, 20 patients (12.8%) needed reinsertion of a drain due to persisting seroma. Within this cohort, 17 patients (85%) needed surgical treatment as definitive therapy, whereas only 3 patients did not require further intervention (*p* = 0.045). Therefore, setting the indication for the reinsertion of a drain in the entire cohort was associated with a 3.3 times higher risk of having surgical revision in the end.

During the follow-up period, seroma recurrence was observed among 17 patients (10.9%). Of these patients with seroma recurrence, two were treated surgically with seroma excision, debridement, and closure over drains anew, whereas the rest were treated conservatively. The patients with seroma recurrence initially had seromas for 63.4 days on average, with durations ranging from 1 to 365 days before initiating the primary surgical treatment.

## 4. Discussion

Seroma formation is a common postoperative complication encountered following a wide variety of surgical procedures, ranging from breast surgery to flap harvesting and body contouring [5,10]. Its relative frequency and often mild presentation of symptoms may cause its occurrence to be considered a minor complication that can be expected after major surgery. However, its course can be complicated by infection through repetitive aspirations, wound healing problems, and even skin necrosis. The sometimes persistent and recurring nature of a seroma, with a prolonged course of management, is often frustrating for both the patient and the surgeon. Therefore, it is important to recognize what risk factors in the usual evolution might lead to revisional surgery.

There is considerable variability in the literature regarding risk factors and stratifications for postoperative seroma formation. While Loo et al. [8] and Burak et al. [9] described increased BMI and hypertension as risk factors for postoperative seroma formation, Gonzalez et al. and Hashemi et al. reported that only the surgery type was significant for influencing postoperative seroma incidence [12,13]. Some authors have proposed large volumes of drainage in the first 3 days as a risk factor for subsequent seroma formation. However, the duration of drainage was not found to have an influence on seroma formation in the studies reported by Kuroi et al. or Iida et al. [14]. Moreover, the available literature on predictors for revisional surgery in already formed seromas is scarce. The only clear recommendations for definitive surgical treatment have been reported in Morel-Lavallée lesions, defined as closed degloving and posttraumatic seromas [15]. Moreover, Nickerson et al. suggested that an aspirated volume of >50 mL should prompt operative intervention in these cases [16]. On examining various patients` characteristics, we found that comorbidities, such as heart failure and coronary heart disease, were associated with a higher need for revisional surgery (*p* < 0.05). However, we did not find the same results when evaluating BMI values or the presence of hypertension in our patients, as previous studies have shown.

Various therapeutic attempts, such as compression dressings and immobilization [17,18], the application of different tissue adhesives (e.g., fibrin sealant) [19], sclerotherapy agents [20,21,22,23], and even a therapeutic attempt using tranexamic acid, have failed to show any benefits for minimizing seromas. Dudai et al. also proposed intraoperative hypertonic saline irrigation as a new therapeutic sclerosant in preventing subcutaneous seroma formation after abdominal wall hernia surgeries, enhancing adhesion formation, and reducing drain secretion [24]. Recent focus has been put on Bioglass/Ceria nanoparticles in the treatment of seromas, with significant results in early seroma reduction having been found within an experimental preclinical setting in a rat model constructed by our group. Yet, the long-term effects of nano-bridging in reducing seroma formation, and the exact mechanism of action behind it, still need to be fully understood before conducting clinical trials [11]. Other non-surgical strategies include serial aspiration and drainage. The concept of drainage was first described in 1947 as a therapeutic measure against seroma formation that facilitated wound healing [25,26]. Having a drain vs. no drain, the suction pressure, the number of drains, and the duration of drainage have been extensively analyzed in the literature on wound drainage [27,28,29]. Here, we demonstrate that the reinsertion of a drain in cases of recurrent seroma formation was significantly associated with a higher rate of surgical revision. This highlights the fact that even though early drain removal reduces the risk of wound complications and is less bothersome for the patients, in cases where there is persistent seroma fluid production, the drains should be left in situ. The early removal of a drain in persistent seroma production may be related to an increased risk of reinsertion and, therefore, may be associated with a higher chance of a definitive secondary surgical treatment [30,31]. Commonly, it is the individual surgeon’s choice when to remove the drain. Usually, this occurs when the postoperative drainage volume is less than 50 mL within 24 h, and this may last up to 1–2 weeks. In our unit, the standard of care is to remove the drains when the draining volume is under 30 mL over 24 h. Loo et al. described that the need for drainage for more than 8 days and drainage output exceeding 500 mL over the course of the first three postoperative days would be associated with an increased risk for postoperative seroma formation [8,32,33,34].

Standard surgical therapeutic options for seromas include debridement, quilting and progressive tension sutures, surgical marsupialization, and excision or debridement of the seroma capsule [35,36]. Seroma may reoccur after the initial resolution—for example, after adjuvant or neoadjuvant therapy—or seroma may remain unresolved or persist over months despite conservative treatment, such as needle aspirations, finally requiring surgical intervention [37]. Our data demonstrate that patients with seromas treated with aspirations for over 40 days had an over 9 times higher risk of requiring revisional surgery compared to patients where the duration of seroma aspiration was under 40 days (*p* = 0.001). Consequently, among these patients, it is highly recommended that revisional surgery be considered sooner rather than later in order to shorten seroma duration overall. Our findings are in line with a previous study conducted by Lee et al. [33,37], where patients with rare cases of refractory seroma over 4 weeks after breast reconstruction with a latissimus dorsi flap, for whom repeated, prolonged needle aspirations were necessary during follow-up, finally required surgical intervention [37].

We acknowledge that this clinical study examining predisposing factors and management concepts in patients with postoperative seroma has limitations. Its retrospective nature means that the variation in treatment practices over the years is a confounding factor, and that some described treatments reported in the literature (instillation of astringent/sclerosing agents in the seroma cavity or transcutaneous external netting) were not included, since they were not employed in our unit. Furthermore, we studied symptomatic postoperative seroma formation in patients who underwent a large variety of primary surgeries, so increased variability is to be expected. Moreover, subclinical, asymptomatic seromas were not included in our study. Nevertheless, our results demonstrate clear indicators for revision surgery during the process of seroma formation, such as the duration of repeated needle aspirations or the need for drain reinsertion.

## 5. Conclusions

Persisting and recurrent seroma formation is a serious postoperative complication with a prolonged management course, which is frustrating for both the patient and the treating surgeon. It is crucial to establish a proper therapeutic approach for seroma and to promptly stratify patients who may suffer from refractory seroma formation and need revisional surgery. Based on our data, patients requiring seroma aspiration should be counseled on surgical treatment sooner rather than later, as prolonged aspiration time (over 40 days) greatly increases the risk of surgical revision. Moreover, the reinsertion of a drain should only be used as a temporizing measure, at most, and patients requiring a drain to control the size of the seroma should promptly be scheduled for surgical revision.

## Figures and Tables

**Table 1:** Patients’ characteristics.

Patients’ Characteristics		Number of Patients (Percentages [%])
Gender	Male	64/156 (41%)
	Female	92/156 (59%)
Comorbidities	History of smoking	146/156 (93.6%)
	History of alcohol consumption	149/156 (95.5%)
	Diabetes mellitus	20/156 (12.8%)
	Arterial hypertension	48/156 (30.8%)
	Heart failure	13/156 (8.3%)
	Coronary heart disease	13/156 (8.3%)
	Impaired coagulation	33/156 (21.2%)
	History of chemotherapy	21/156 (13.4%)
	History of radiotherapy	34/156 (21.8%)
Prophylactic measures against seroma *	No	70/156 (44.8%)
	Yes	86/156 (55.2%)
Seroma location	Lower extremity	56/156 (35.9%)
	Inguinal area	30/156 (19.2%)
	Posterior trunk	19/156 (12.1%)
	Abdomen	18/156 (11.5%)
	Breast	17/156 (10.9%)
	Axillary area	12 (7.7%)
	Other	4/156 (2.5%)
Aspiration performed	Yes	64/156 (41%)
	No	92/156 (59%)
Reinsertion of drain	Yes	20/156 (12.8%)
	No	136/156 (87.5%)
Wound-related readmission	Yes	29/156 (18.6%)
	No	127/156 (81.4%)
Surgical definitive treatment	Yes	103/156 (66%)
	No	53/156 (34%)

* Prophylactic measures against seroma: drains, fibrin glue, external compression, quilting sutures.

**Table 2:** Patients’ characteristics.

Patients’ Characteristics	Median (Range)
Age [years]	58 (17–93)
BMI [kg/m^2^]	26.5 (15.2–65)
Postoperative drain output until removal [mL]	597 (4–9925)
Time until last drain removed postoperatively [days]	7 (1–32)
Seroma duration [days]	45 (3–1218)
Number of aspirations per patient	2 (1–9)
Aspiration volume *	191 (5–1650)
Number of surgeries per patient	2 (1–8)

* All aspirations of all patients included, some patients underwent up to eight aspirations.

**Table 3:** Seroma treatment during the initial revisional surgery.

Procedure	Number of Patients (%)
Primary definitive treatment after seroma excision and debridement	
Wound closure over drains	32/103 (31.1%)
Defect coverage with a flap	9/103 (8.7%)
Skin graft	1/103 (1.0%)
Delayed closure after seroma excision and debridement	
Negative pressure wound therapy	46/103 (44.7%)
Wound healing by secondary intention	15/103 (14.6%)

## Data Availability

The data presented in this study are available upon request from the corresponding author.
